# Reverse shock index multiplied by Glasgow Coma Scale (rSIG) predicts mortality in severe trauma patients with head injury

**DOI:** 10.1038/s41598-020-59044-w

**Published:** 2020-02-07

**Authors:** Chu Wan-Ting, Liao Chin-Hsien, Lin Cheng-Yu, Chien Cheng-Yu, Lin Chi-Chun, Chang Keng-Wei, Chen Jiann-Hwa, Chen Wei-Lung, Huang Chien-Cheng, Lim Cherng-Jyr, Chung Jui-Yuan

**Affiliations:** 1 Department of Emergency Medicine, Ton-Yen General Hospital, Hsinchu county, Zhubei City, Hsinchu county, Taiwan; 2 Department of Emergency Medicine, Cathay General Hospital, Taipei, Taiwan; 3 Department of Emergency Medicine, Chang Gung Memorial Hospital, and Chang Gung University College of Medicine, Linkou, Taiwan; 4 Fu Jen Catholic University School of Medicine, Taipei, Taiwan; 5 Department of Emergency Medicine, Chi-Mei Medical Center, Tainan, Taiwan; 6 Department of Environmental and Occupational Health, College of Medicine, National Cheng Kung University, Tainan, Taiwan; 7 Department of Senior Services, Southern Taiwan University of Science and Technology, Tainan, Taiwan

**Keywords:** Risk factors, Risk factors, Trauma, Risk factors, Trauma

## Abstract

The reverse shock index (rSI), a ratio of systolic blood pressure (SBP) to heart rate (HR), is used to identify prognosis in trauma patients. Multiplying rSI by Glasgow Coma Scale (rSIG) can possibly predict better in-hospital mortality in patients with trauma. However, rSIG has never been used to evaluate the mortality risk in adult severe trauma patients (Injury Severity Score [ISS] ≥ 16) with head injury (head Abbreviated Injury Scale [AIS] ≥ 2) in the emergency department (ED). This retrospective case control study recruited adult severe trauma patients (ISS ≥ 16) with head injury (head AIS ≥ 2) who presented to the ED of two major trauma centers between January 01, 2014 and May 31, 2017. Demographic data, vital signs, ISS scores, injury mechanisms, laboratory data, managements, and outcomes were included for the analysis. Logistic regression and receiver operating characteristic analysis were used to evaluate the accuracy of rSIG score in predicting in-hospital mortality. In total, 438 patients (mean age: 56.48 years; 68.5% were males) were included in this study. In-hospital mortality occurred in 24.7% patients. The median (interquartile range) ISS score was 20 (17–26). Patients with rSIG ≤ 14 had seven-fold increased risks of mortality than those without rSIG ≤ 14 (odds ratio: 7.64; 95% confidence interval: 4.69–12.42). Hosmer–Lemeshow goodness-of-fit test and area under the curve values for rSIG score were 0.29 and 0.76, respectively. The sensitivity, specificity, positive predictive value, and negative predictive values of rSIG ≤ 14 were 0.71, 0.75, 0.49, and 0.89, respectively. The rSIG score is a prompt and simple tool to predict in-hospital mortality among adult severe trauma patients with head injury.

## Introduction

Trauma, the sixth leading cause of death worldwide and a major cause of morbidity and mortality, includes hemorrhagic shock and traumatic brain injuries^1–4^. Head injuries are frequent associated with trauma; approximately 1.4 million emergency department (ED) visits, 275,000 hospitalizations, and 52,000 deaths associated with head injuries have been in the United States each year^5^. Trauma patients with a higher Injury Severity Score (ISS) and a greater frequency of transfusions are at increased risk of in-hospital mortality within the first 24 hours^6^. Therefore, it is important to identify trauma patients with high mortality risk and commence aggressive resuscitation and proper medical intervention.

The Shock index (SI), defined as the ratio of heart rate (HR) to systolic blood pressure (SBP), was developed by Allgower and Burri in 1967; it has been used to identify trauma patients with hypovolemic shock^7^. According to previous studies, SI ≥ 1 is indicative of an uncompensated shock status and is associated with a higher mortality rate^8–10^. However, practitioners generally view unstable hemodynamic status as SBP lower than HR and not as HR higher than SBP. Therefore, a research group in Taiwan introduced the concept of reverse (or inverse) shock index (rSI), defined as the ratio of SBP to HR and reported that rSI <1 was associated with poor outcome and may help identify trauma patients at high risk of mortality even without hypotension^11–14^.

The Glasgow Coma Scale (GCS)^15^, which is used to assess consciousness level, has also shown to possess strong correlation with mortality in patients with traumatic brain injury^16,17^. Considering these characteristics, a Japanese research group has proposed a new scoring tool, rSIG, which was derived from a multicenter retrospective study and calculated by multiplying rSI by GCS score^18^. They found that the rSIG score can discriminate in-hospital mortality risk and is as good as the previous prediction methods that used only vital signs and age^18^.

However, rSIG has never been used to evaluate the mortality risk in adult severe trauma patients (ISS ≥ 16) with head injury (head Abbreviated Injury Scale [AIS] ≥ 2) in the ED. Therefore, this study aimed to investigate the predictive performance of rSIG for in-hospital mortality in adult severe trauma patients with head injury.

## Results

Overall, 438 patients (aged [mean ± SD]: 56.48 ± 21.06 years; 68.5% males) were included in this study. The in-hospital mortality rate was 24.7% (Table 1). The ISS, AIS, ward, and intensive care unit (ICU) stay were not normally distributed and were, therefore, displayed by median (IQR). The median (IQR) of ISS and AIS was significantly higher in the mortality group than in the survival group (25^11,17–28^ and 16^16–25^ vs 18^16–25^ and 16^9–16^, respectively). Traffic accident (45.3%) was the most common injury mechanism, with motorcycle accidents accounting for 34.7% despite wearing helmets according to the Taiwan traffic regulations. Fall from > 2 meters (18.9%) and falling down (17.6%) were the second and third common injury mechanisms; both significantly higher in the mortality group than in the survival group.

**Table 1:** Characteristics of severe trauma adult patients (ISS > 16) with head injury (head AIS ≥ 2) in the ED.

Characteristics	Total patients (n = 438)	Survival (n = 330)	Mortality (n = 108)	*p*-value
Age	56.48 ± 21.06	55.61 ± 21.55	59.15 ± 19.33	0.13
Sex (Male)	68.5	25.0	75.0	0.04
Triage	1.95 ± 0.79	2.16 ± 0.74	1.32 ± 0.62	<0.01
ISS score	20 (17–26)	18 (16–25)	25 (17–29)	<0.01
Head	16 (16–16)	16 (9–16)	16 (16–25)	0.02
**Injury mechanism**				
Fall from >2 meters	18.9	16.1	27.8	<0.01
Assault	0.9	1.2	0	0.25
Suicide	0.5	0	1.9	0.01
Falling down	17.6	21.5	5.6	<0.01
Others	16.2	16.7	14.8	0.65
**Traffic accident**				
Motorcycle rider	34.7	35.8	31.5	0.42
Car driver	3.4	2.7	5.6	0.16
Bicycle rider	1.1	1.2	0.9	0.81
Pedestrian	6.6	4.8	12.0	<0.01
**Vital signs**				
SBP (mmHg)	144.94 ± 39.97	147.04 ± 33.38	138.34 ± 55.11	0.04
DBP (mmHg)	80.75 ± 20.91	81.0 ± 17.85	71.99 ± 32.23	0.01
Heart rate (beats/min)	88.42 ± 56.81	91.22 ± 62.15	79.66 ± 33.90	0.02
Body temperature (°C)	35.99 ± 3.55	36.25 ± 2.06	35.15 ± 6.22	<0.01
Respiratory rate (min)	19.11 ± 8.34	19.52 ± 7.37	17.80 ± 10.80	0.07
SpO2 (%)	92.68 ± 19.72	95.98 ± 12.10	79.73 ± 34.00	<0.01
GCS score	11.00 ± 6.01	12.70 ± 5.19	6.28 ± 4.25	<0.01
**Past History**				
Hypertension	22.6	19.7	31.5	0.01
Diabetes Mellitus	12.6	11.2	16.7	0.13
Heart disease^†^	3.7	3.9	2.8	0.58
COPD	1.1	0.6	2.8	0.06
Cancer	0.9	0.6	1.9	0.24
Liver cirrhosis	1.4	0.9	2.8	0.15
Chronic kidney disease	0.9	0.6	1.9	0.24
**Scores**				
SI	0.61 ± 0.51	0.64 ± 0.55	0.53 ± 0.35	0.02
rSI	1.77 ± 1.37	1.79 ± 0.52	1.72 ± 0.81	0.55
rSIG	19.49 ± 18.10	22.29 ± 19.43	10.92 ± 8.88	<0.01
**Laboratory data**				
WBC (10^3^ cells/mm^3^)	11.61 ± 10.03	11.49 ± 11.24	11.99 ± 4.95	0.53
Hemoglobin (g/dL)	13.94 ± 8.93	14.33 ± 10.19	12.74 ± 2.34	<0.01
Platelet (10^3^/mm^3^)	214.17 ± 86.02	219.52 ± 79.21	198.51 ± 102.23	0.03
PT (seconds)	10.48 ± 1.62	10.20 ± 1.19	11.30 ± 2.29	<0.01
aPTT (seconds)	27.51 ± 14.83	25.89 ± 14.82	32.25 ± 13.88	<0.01
Glucose (mg/dL)	168.00 ± 132.00	160.32 ± 135.04	190.81 ± 120.26	0.03
**Management**				
Whole body CT	26.5	16.1	58.3	<0.01
Intubation	35.8	18.5	81.5	<0.01
Chest tube	2.3	1.5	4.6	0.06
Blood transfusion	9.6	3.8	27.8	<0.01
Admission^*^				
Ward days	7 (1–14)	9 (4–15)	0 (0–0)	<0.01
ICU days	3 (0–6)	3 (0–6)	3 (1–6)	0.01

^†^Heart disease includes coronary artery disease and congestive heart failure.

^*^Admission to general ward or intensive care unit.

Data were presented as %. Data with normal distribution were displayed as mean ± standard deviation. Data that were not normally distributed, will be displayed as median (interquartile range). ISS, Injury Severity Score; AIS, Abbreviated Injury Scale; SBP, Systolic blood pressure; DBP, Diastolic blood pressure; SpO_2_, Saturation of peripheral oxygen; GCS, Glasgow coma scale; COPD, Chronic obstructive pulmonary disease; SI, Shock index; rSI, Reverse Shock Index; rSIG, Reverse Shock Index multiplied by Glasgow coma scale; WBC, White blood cell; PT, Prothrombin Time; aPTT, Activated Partial Thromboplastin Time; BUN, Blood Urea Nitrogen; GOT, Aspartate Aminotransferase; GPT, Alanine Aminotransferase; CT, computer tomography; ICU, Intensive care unit.

The mean ± SD of SBP, HR, and GCS score were 144.94 ± 39.97 mmHg, 88.42 ± 56.81 per minute, and 11.00 ± 6.01, respectively. SBP and HR were lower in the mortality group (138.34 ± 55.11 mmHg and 79.66 ± 33.90 per minute, respectively) than in the survival group (147.04 ± 33.38 mmHg and 91.22 ± 62.15 per minute, respectively). The mortality group had significantly lower GCS score (6.28 ± 4.25) than the survival group (12.70 ± 5.19; *p* < 0.01). In addition, significantly higher prevalence of hypertension was noted in the mortality group than in the survival group.

Laboratory data analysis showed that mortality group had higher prothrombin time (PT), activated partial thromboplastin time (aPTT), and glucose level than the survival group. Platelet counts were lower in the mortality group than in the survival group. The rSIG score was 10.92 ± 8.88 and 22.29 ± 19.43 in the mortality and survival groups, respectively (p < 0.01). The rSI and shock index (SI) were both lower in the mortality group for 1.72 ± 0.81 and 0.53 ± 0.35. The percentage of whole-body CT, endotracheal tube intubation, and blood transfusion was higher in the mortality group than in the survival group. None of the study patients were discharged from the ED. Patients in the survival group had longer ward stay (9^4–15^ days), while those in the mortality group had a longer ICU stay (3^1–6^) days.

**Figure 1:**
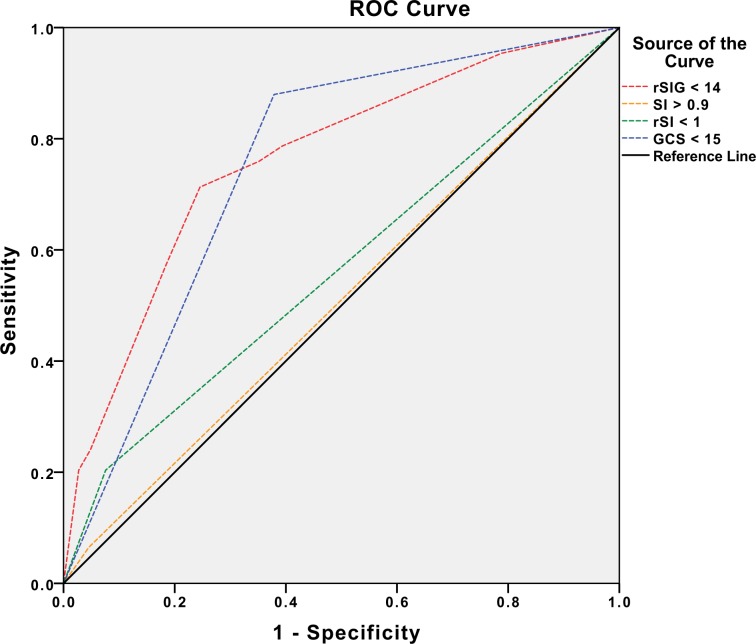
Area under the curve of rSIG < 14, SI > 0.9, rSI < 1, and GCS < 13. rSIG, Reverse shock index multiplied by Glasgow Coma Scale, SI, Shock index; rSI, Reverse shock index; GCS, Glasgow Coma Scale.

The best cut-off level of rSIG score to predict mortality in adult severe trauma patients with head injury was 14, analyzed via the Youden Index. The mortality predictive ability of rSIG < 14 was further assessed via logistic regression and compared with SI > 0.9, rSI < 1, and GCS < 13 (cut-off point calculated via Youden index). The results showed that severe trauma patients with rSIG < 14 had the highest risk of mortality for 7.64-fold, while GCS < 13 was 6.16-fold. (Table 2). AUROC was performed as well and adjusted by sex (*p* = 0.04), and hypertension (*p* = 0.01). The adjusted AUROC for mortality prediction showed both rSIG < 14 and GCS < 13 had acceptable mortality discrimination ability for 0.76 (0.71–0.82) and 0.74 (0.70–0.80), (Table 3, Fig. 1). The mortality prediction performance of rSIG < 14 in adult severe trauma patients with head injury showed sensitivity of 0.71 (95% CI 0.68–0.74), specificity of 0.75 (95% CI 0.74–0.77) and negative predictive rate of 0.89 (95% CI 0.87–0.90) (Table 4).

**Table 2:** Mortality rate prediction comparison between rSIG < 14, SI > 0.9, rSI < 1, and GCS < 13, identified by logistic regression.

	Odds ratio	95% CI	p-Value	Hosmer–Lemeshow goodness of fit
rSIG < 14	7.64	4.69–12.42	<0.01	0.29
SI > 0.9	0.46	0.13–1.60	0.22	—
rSI < 1	1.89	0.78–4.59	0.16	—
GCS < 13	6.16	3.01–12.63	<0.01	—

rSIG, Reverse shock index multiplied by Glasgow Coma Scale; SI, Shock index; rSI, Reverse shock index; GCS, Glasgow Coma Scale.

**Table 3:** AUROC for mortality discrimination of rSIG < 14, SI > 0.9, rSI < 1, and GCS < 13, in severe trauma adult patients (ISS ≥ 16) with head injury (head AIS ≥ 2), adjusted by sex and hypertension.

	AUROC	95% CI	p Value
rSIG < 14	0.76	0.71–0.82	<0.01
SI > 0.9	0.51	0.45–0.57	0.76
rSI < 1	0.56	0.49–0.63	0.05
GCS < 13	0.74	0.70–0.80	<0.01

AUROC, Area under the curve; ISS, Injury Severity Score; AIS, Abbreviated Injury Scale; rSIG, Reverse shock index multiplied by Glasgow Coma Scale; SI, Shock index; rSI, Reverse shock index; GCS, Glasgow Coma Scale; CI, Confidence interval

**Table 4:** Performance of rSIG < 14 in predicting mortality in severe trauma adult patients (ISS ≥ 16) with head injury (head AIS ≥ 2).

Performance	rSIG < 14
Sensitivity	0.71 (0.61–0.79)
Specificity	0.75 (0.71–0.80)
Positive predictive value	0.49 (0.43–0.54)
Negative predictive value	0.89 (0.85–0.91)

rSIG, Reverse shock index multiplied by Glasgow Coma Scale; ISS, Injury Severity Score; AIS, Abbreviated Injury Scale.

## Discussion

Due to the increased mortality risks in adult trauma patients, several prediction models have been developed for mortality prediction, such as Trauma and Injury Severity Score (TRISS) and ISS. TRISS is frequently used to predict survival probabilities and has the best accuracy thus far. It comprises ISS, RTS, age, and the mechanism of injury^19,20^. However, it involves complicated equations and calculations as the RTS is a physiological score consists of the weighted summation of coded GCS score, RR, and SBP, calculated by the following formula: RTS = 0.9368 GCS + 0.7326 SBP + 0.2908 RR, which is impractical for real-time management of trauma patients in the ED^21^. Similarly, ISS score uses anatomical variable to grade the severity of trauma patients by the summation of squares of AIS score in the three most severe injured body regions of six predefined body territories^22^. All these models require coded scoring systems that are difficult to remember.

Trauma patients with severe injuries often have concurrent head injury. These complicate management and are associated with higher in-hospital mortality. Therefore, a quick and easy tool for real-time risk stratification due to the dynamic change during management of these patients is needed. The rSIG uses easily obtainable physiological variables (SBP, HR, and GCS) and is simple to calculate. A study conducted by SC Wu *et al*. showed the best rSIG cutoff point was 14.8 for trauma patients with head injury, with 86.8% sensitivity and 70.7% specificity^23^. Similarly, our study found that rSIG is a useful tool to predict mortality in adult severe trauma patients with head injury. However, the best rSIG cutoff point was 14 in our study population, due to the significantly lower GCS score (6.28 ± 4.25) in the mortality group than in the survivor group (12.70 ± 5.19).

The mortality predictive strength of SI > 0.9, rSI < 1, and GCS < 13 among adult severe trauma patients with head injury (head AIS ≥ 2) were also analyzed. GCS < 13 had the greatest risk of mortality for 6.16-fold; while rSI and SI were 1,89-fold and 0.46-fold. After multiplying rSI and GCS, and discovering the best cut-off point, the AUROC of rSIG < 14 was calculated as 0.76, after adjusting for sex and hypertension. The negative predictive rate was 0.89, making rSIG an effective tool to rule out mortality in adult severe trauma patients with head injury (head AIS ≥ 2) who scored ≥ 14.

Although older patients tend to have higher baseline SBP even after injury, which may probably underestimate the severity of underlying shock in older traumatized patients^24^, Zarzaur *et al*. proposed a solution by multiplying SI by age (SIA)^25^. In another study, Kimura *et al*. compared the performance of rSIG and rSIG multiplied by age (rSIG/A). They found that rSIG had slightly better survival discrimination ability than rSIG/A with AUROC calculated as 0.90 and 0.88 respectively, in younger trauma patients aged less than 55 years. Meanwhile, in older trauma patients aged 55 years and older, both rSIG and rSIG/A had similar survival discrimination ability with the calculated AUROC being 0.84 and 0.83, respectively^18^. Hence, rather than multiplying rSIG/A, it is reasonable to use rSIG in our study for simplification and easy applicability.

This is the first study to report the utility of rSIG score for mortality prediction among severe adult trauma patients with head injury. However, it has some limitations. First, key data and information was missing due to the retrospective nature of this study. Second, the complexity of trauma patients may be different in other centers as this study was conducted in two trauma centers. Third, this study includes mostly traffic accident and blunt trauma patients, thus, the result and cutoff values may not be applicable to other settings and further validations in other patient populations in different settings are required. Fourth, the vital signs were obtained only once while arriving at the ED. Although no further sets of vital signs were available, the initial vital signs may reflect the original patient status rather than vital signs obtained after treatment. Finally, the confidence interval of the rSIG < 14 odds ratio was very wide, which indicate a larger sample sized study is warranted to validate the result of this study.

## Methods

### Study design, setting, and participants

This study was conducted in two trauma centers. Cathay General Hospital was a university-affiliated medical center, which consists of 800 ward beds and 40 ED beds. Approximately 55,000 patients visit the ED annually, of which 30% are trauma patients. The other trauma center, Ton-Yen General Hospital has 450 ward beds and 15 ED beds, with about 53,000 trauma patients visiting the ED each year. Adult patients aged ≥18 years with ISS ≥ 16 and head AIS ≥ 2 who presented to the ED of the two trauma centers between January 01, 2014 and May 31, 2017 were included in the study.

### Variable and primary outcome definition

ISS was calculated by dividing the body into the following six AIS regions: head or neck, face, chest, abdominal or pelvic contents, extremities or pelvic girdle, and external. The sum of the squares of the three highest AIS scores among the most severely injured body territories were calculated. There three exceptions for the calculation rule as follows: 75 points should be assigned when any single AIS region scored 6 points; an AIS of 9 should be given if the severity of an injury region cannot be determined; and if the ISS is unable to calculate, 99 points should be assigned^26^.

Shock index (SI) was calculated as HR divided by SBP. According to several previous articles, SI > 0.9 was related with worse outcome in critically ill patients^27^. The rSI was calculated as SBP divided by HR^18^. rSI < 1 was associated with poor prognosis including hospital length of stay and mortality in trauma patients^11^. Subsequently, the rSIG score was measured by multiplying the rSI with GCS. Patients who survived the entire hospitalization course and were discharged successfully were considered “survivors.”

### Data collection and case and control group assignments

**Figure 2:**
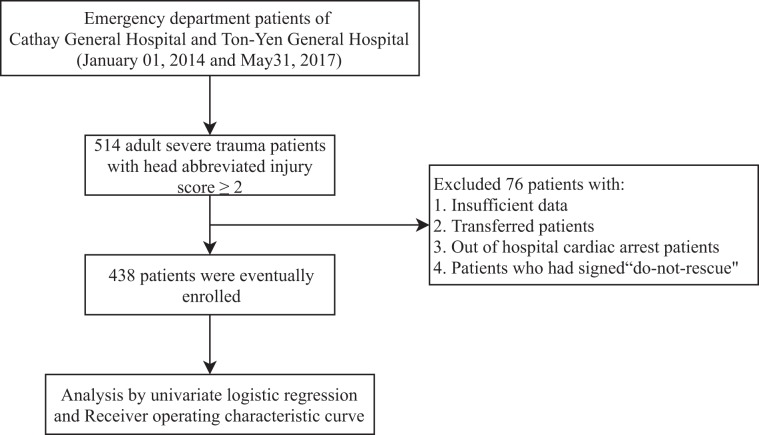
Flowchart of this study.

Adult ED trauma patients who fit the inclusion criteria of ISS score ≥ 16 points and head AIS ≥ 2 were assembled via a retrospective chart review. In total, 514 adult trauma ED patients met the criteria, and patients’ information including demographic characteristics, vital signs, past histories, laboratory data, ISS scores, head AIS, injury mechanisms, management, admission status, and in-hospital mortality were obtained by an emergency physician. The vital signs and laboratory data were obtained immediately upon arrival at the ED. All of the patients were sent to the ED via an ambulance, and the median time (IQR) from response to transport to ED was about 24 (18–30) minutes. According to Chien CY *et al*., the median emergency medical service (EMS) response time to suspect trauma OHCA call in Taiwan Taoyuan district was 6.0 (4.0–8.0); Duration to scene time was 10.0 (8.0–14.0); and transport time from the scene to ED was 6.0 (3.0–9.0)^28^. After excluding 76 patients, 26 patients with insufficient data, 10 transferred patients who had been treated in other hospitals, 20 out of hospital cardiac arrest patients, and 20 patients who had signed “do-not-rescue”, 438 patients were eventually included (Fig. 2). Required data that is not recorded in the patient’s medical chart was considered negative and was excluded. The proportions of patients with insufficient data, was approximately 3.9%, which was below the rule of thumb 5%, therefore, further adjustment for missing data was not necessary^29^. The included patients were then divided into the survival group and the mortality group. All of the variables were used to compare between the two groups (Table 1).

### Ethical statement

This study was approved by the Institutional Review Board of the Cathay General Hospital and was conducted according to the tenets of the Declaration of Helsinki. As the current study was an observational study, the Cathay General Hospital Institutional Review Board approved the study protocol and waived the need for informed consent (written and oral) from the participants.

### Statistical analysis

All statistical analyses were performed using SPSS 23.0 for Mac (IBM Corp., Chicago, IL, USA). The power of this study was calculated for 1.0 via G power 3.0. Normal distribution continuous data are presented as means ± standard deviation (SD). Whereas continuous data that are not normally distributed were summarized as median (interquartile range [IQR]). Independent samples t-test or the Mann–Whitney–Wilcoxon test was used for continuous variables in the univariate analysis. Pearson’s chi-squared test or Fisher’s exact test was used for categorical variables. Mortality prediction of rSIG score in adult severe trauma patients was analyzed via logistic regression. The best cut-off point of rSIG score for mortality prediction in severe adult trauma patients with head injury was calculated by Youden index. The mortality discrimination ability of the rSIG score was identified by using the area under the receiver operating characteristic curve (AUROC) and was adjusted potential confounders that could affect mortality (*p*-value < 0.1) via regression modeling. The Hosmer–Lemeshow goodness-of-fit test was also performed to evaluate the reliability of the scoring systems. The performance of rSIG score for mortality prediction in severe adult trauma patients with head injury, including sensitivity, specificity, positive predictive value, and negative predictive value, were also calculated. Mortality predictive ability between Glasgow Coma Scale (GCS) < 13, the best cut-off value identified by Youden index; Shock index >0.9; Reverse shock index <1; and Reverse shock index multiplied by Glasgow Coma Scale (rSIG) < 14 were compared via logistic regression and AUROC.

## Conclusion

rSIG < 14 is a simple and prompt tool to predict mortality in adult severe trauma patients with head injury (head AIS ≥ 2). It may also assist physicians in dispositioning the patients accurately, and proper medical resources utilization. Mortality could be rule out in adult severe trauma patients with head injury (head AIS ≥ 2) who scored ≥14. Further researches should be conducted to validate the result of this study.

## References

[1] Johansson PI, Stensballe J, Ostrowski SR (2012). Current management of massive hemorrhage in trauma. Scand J Trauma Resusc Emerg Med..

[2] Shere-Wolfe RF, Galvagno SM, Grissom TE (2012). Critical care considerations in the management of the trauma patient following initial resuscitation. Scand J Trauma Resusc Emerg Med..

[3] Cheng P (2017). Trends in traumatic brain injury mortality in China, 2006–2013: A population-based longitudinal study. PLoS Med..

[4] Taylor CA, Bell JM, Breiding MJ, Xu L (2017). Traumatic Brain Injury-Related Emergency Department Visits, Hospitalizations, and Deaths - United States, 2007 and 2013. MMWR Surveill Summ..

[5] Faul, M., Xu, L., Wald M. M. & Coronado, V. G. Traumatic Brain Injury in the United States: Emergency Department Visits, Hospitalizations and Deaths 2002-2006 (Blue Book). *Centers for disease control and prevention*, https://www.cdc.gov/traumaticbraininjury/pdf/blue_book.pdf (2010)

[6] Lefering R (2012). Epidemiology of in-hospital trauma deaths. Eur J Trauma Emerg Surg..

[7] Allgöwer M, Burri C (1967). [“Shock index”]. Dtsch Med Wochenschr..

[8] Rady MY, Nightingale P, Little RA, Edwards JD (1992). Shock index: a re-evaluation in acute circulatory failure. Resuscitation..

[9] Sloan EP, Koenigsberg M, Clark JM, Weir WB, Philbin N (2014). Shock index and prediction of traumatic hemorrhagic shock 28-day mortality: Data from the DCLHb resuscitation clinical trials. West J Emerg Med..

[10] Mitra B, Fitzgerald M, Chan J (2014). The utility of a shock index ≥ 1 as an indication for pre-hospital oxygen carrier administration in major trauma. Injury..

[11] Chuang JF (2016). Use of the reverse shock index for identifying high-risk patients in a five-level triage system. Scand J Trauma Resusc Emerg Med..

[12] Kuo SCH (2016). The use of the reverse shock index to identify high-risk trauma patients in addition to the criteria for trauma team activation: a cross-sectional study based on a trauma registry system. BMJ Open..

[13] Lai WH (2016). Using the Reverse Shock Index at the Injury Scene and in the Emergency Department to Identify High-Risk Patients: A Cross-Sectional Retrospective Study. Int J Environ Res Public Health..

[14] Lai WH (2016). Systolic Blood Pressure Lower than Heart Rate upon Arrival at and Departure from the Emergency Department Indicates a Poor Outcome for Adult Trauma Patients. Int J Environ Res Public Health..

[15] Teasdale G, Jennett B (1974). Assessment of coma and impaired consciousness. A practical scale. Lancet..

[16] Emami P (2017). Impact of Glasgow Coma Scale score and pupil parameters on mortality rate and outcome in pediatric and adult severe traumatic brain injury: A retrospective, multicenter cohort study. J Neurosurg..

[17] Nik A (2018). The Efficacy of Glasgow Coma Scale (GCS) Score and Acute Physiology and Chronic Health Evaluation (APACHE) II for Predicting Hospital Mortality of ICU Patients with Acute Traumatic Brain Injury. Bulletin of emergency and trauma..

[18] Kimura A, Tanaka N (2018). Reverse shock index multiplied by Glasgow Coma Scale score (rSIG) is a simple measure with high discriminant ability for mortality risk in trauma patients: An analysis of the Japan Trauma Data Bank. Crit Care..

[19] Gabbe BJ, Cameron PA, Wolfe R (2004). TRISS: Does it get better than this?. Acad Emerg Med..

[20] de Munter L (2017). Mortality prediction models in the general trauma population: A systematic review. Injury..

[21] Champion HR (1989). A revision of the Trauma Score. J. Trauma..

[22] Baker SP, O’Neill B, Haddon W, Long WB (1974). The injury severity score: A method for describing patients with multiple injuries and evaluating emergency care. J. Trauma..

[23] Wu SC (2018). The Reverse Shock Index Multiplied by Glasgow Coma Scale Score (rSIG) and Prediction of Mortality Outcome in Adult Trauma Patients: A Cross-Sectional Analysis Based on Registered Trauma Data. Int. J Environ. Res Public Health..

[24] Zarzaur BL, Croce MA, Fischer PE, Magnotti LJ, Fabian TC (2008). New vitals after injury: shock index for the young and age x shock index for the old. J. Surg. Res..

[25] Zarzaur BL, Croce MA, Magnotti LJ, Fabian TC (2010). Identifying life-threatening shock in the older injured patients an analysis of the national trauma data bank. J. Trauma..

[26] Stevenson M, Segui-Gomez, Lescohier I, Di Scala C, McDonald-Smith G (2001). An overview of the injury severity score and the new injury severity score. Inj Prev..

[27] Cannon CM (2009). Utility of the shock index in predicting mortality in traumatically injured. J. Trauma..

[28] Chien CY (2016). Is 15 minutes an appropriate resuscitation duration before termination of a traumatic cardiac arrest? A case-control study. Am. J. Emerg. Med..

[29] Jakobsen JC, Gluud C, Wetterslev J, Winkel P (2017). When and how should multiple imputation be used for handling missing data in randomised clinical trials - a practical guide with flowcharts. BMC Med. Res. Methodol..

